# The need for better analysis of observational studies in orthopedics: A retrospective study of elbow fractures in children

**DOI:** 10.3109/17453674.2010.487243

**Published:** 2010-05-21

**Authors:** Per-Henrik Randsborg, Einar A Sivertsen, Inge Skråmm, J rat Šaltyt Benth, Pål Gulbrandsen

**Affiliations:** ^1^Department of Orthopedic Surgery; ^2^HØKH Research Centre, Akershus University Hospital, Lørenskog; ^3^Faculty Division, University of Oslo, Akershus University Hospital, OsloNorway

## Abstract

**Background and purpose:**

The conventional statistical methods employed in observational studies in orthopedics require the fundamental assumption that the outcomes are independent. However, fractures treated by the same surgeon cannot be regarded as being independent of each other and should be nested in the statistical analysis. If the effect on outcome of early rather than delayed surgery depends on the severity of the fracture, we have a case of interaction. This is rarely considered in orthopedic research, but could affect the conclusions drawn. The aim of this paper is to describe the concepts of multilevel modeling and interaction in orthopedics.

**Patients and methods** In a cohort of 112 patients with single supracondylar humerus fractures, 78 patients were examined clinically on average 4 years after surgery. The range of motion was measured and the global satisfaction was assessed. The results were used to compare traditional least-squares regression analysis with a 2-level model with interactions.

**Results:**

We found that 25% of the variance in outcome could be attributed to between-surgeon variance. We identified an interaction between the surgeons' experience and the severity of the fractures that influenced the conclusions. The variable “number of pins” was not significant in the 2-level model (p = 0.07), while the ordinary least-squares analysis gave a result that was statistically significant (p = 0.01).

**Interpretation:**

Researchers should consider the need for a 2-level model and the presence of interactions. Standard statistical methods might lead to wrong conclusions.

## Introduction

Supracondylar humerus fractures are the most common elbow injuries in children who require surgery. Percutanous pinning has become the method of choice in most clinics, and severe complications are rare (Otsuka and Kasser 1997). Recent reports have suggested that delay of surgery until the next day is safe (Iyengar et al. 1999, Mehlman et al. 2001, Leet et al. 2002, Gupta et al. 2004). However, vascular injuries and compartment syndromes still occur (Ramachandran et al. 2008), and some authors recommend treating these fractures as early as possible (Walmsley et al. 2006). The statistical methods employed in these publications are t-test, the chi-square test, and simple and multiple regression analyses. These methods require the fundamental assumption that the observed outcomes are independent, which implies that none of the fractures should have been operated by the same surgeon, and the distribution of fractures among surgeons should be random. This is rare, if not impossible, in observational studies on orthopedic surgery.

In the social sciences, there has been a gradual awareness of the inability of conventional statistical methods to analyze the complexity of human interaction. Researchers in public health have repeatedly encouraged the integration of social science methods in medical research (Singer and Ryff 2000, Office of Behavioral and Social Sciences Research 2001). For instance, we should not consider a priori that there is no correlation between outcomes of patients who are operated by the same surgeon. This is not simply a matter of controlling for the experience of the surgeon. Regardless of the ability of the surgeon, the results for fractures treated by the same hands are likely to be more similar than if they were not. Ignoring such correlations may lead to underestimation of standard errors, increasing the risk of committing a type-I error with the conclusion that a variable is statistically significant when it is not. A multilevel approach (also called hierarchical modeling) accounts for potential correlations by modeling intercepts and regression coefficients as random. The intraclass correlation coefficient (ICC) expresses the amount of dependency among observations and is calculated to decide whether a multilevel analysis is appropriate. The ICC can take values from 0 to 1. A non-zero value of ICC implies that the observations are not uncorrelated and that there is a need for multilevel modeling.

Furthermore, orthopedic researchers should consider the existence of interaction, also known as effect modification (Moyé 2006). An interaction is defined as a factor that modifies the independent factor under study. This is analytically more complex than simple confounding. A confounder has the same effect on outcome for all values of the other independent variables studied. Interactions reflect that the effect of one variable depends on the values of one or more other variables. For example, the influence of the surgeon's experience on outcome could be stronger for severe fractures than for less complicated ones. In such cases, a statistical model with interactions should be tested.

Here we describe to the orthopedic community the concept of multilevel modeling and interactions as necessary statistical tools in observational studies. We show that the conventional statistical methods currently employed in retrospective reports may yield misleading results.

## Patients and methods

The study protocol was approved by the Regional Ethics Committee on October 25, 2007 (registration number 1.2007.2093). The patient population consisted of all children who underwent reduction and pinning of a displaced supracondylar humerus fracture in our institution between 1999 and 2006. The patients were identified in our computer files and the medical records were examined. Patients treated with closed reduction without pinning (n = 14) and patients treated at different institutions (n = 6) were excluded, as well as 1 patient with bilateral fractures. We included 112 supracondylar fractures in the study (Table 1). The child's age, sex, preoperative soft tissue and neurovascular status, time of injury, time to surgery, configuration of pins, time to pin removal, any complications, and postoperative status were recorded. Surgeons were classified in two groups according to their level of experience: consultants and residents. If two surgeons performed an operation together, the most experienced was chosen as being responsible for treatment of that fracture.

**Table 1.** Demographic and fracture characteristics of 112 supracondylar humerus fractures in 112 children

Characteristic	Total
No. of fractures	112
Age, mean (range) SD	6.1 (1.6–12.4) 2.1
Males, n (%)	62 (55)
Left side, n (%)	69 (62)
Gartland 2, n (%)	47 (42)
Gartland 3, n (%)	65 (58)
Pulseless, n (%)	5 (4)
cold hand	2 (2)
warm hand	3 (3)
Weak pulse, n (%)	2 (2)
Reduced sensation, n (%)	6 (5)
Radial	0
Ulnar	1
Median	5
Open or threatened skin, n (%)	3 (3)
Open	2 (2)
Threatened skin	1 (1)
Injury–surgery time (h), median (range) SD	7.5 (1.5–190) 19.7
Pin configuration, n (%)	
2 lateral	1 (1)
2 crossed	75 (67)
2 lateral, 1 medial	29 (26)
2 lateral, 2 medial	6 (5)
Hospitalization time (days), median (range) SD	1.0 (1–7) 0.8
Time to pin removal (days), median (range) SD	32 (20–51) 5.6
Night operations, n (%)	20 (18)
Main surgeons, n (%)	30
Residents	10 (33)
Consultants	20 (67)

### Radiographic analysis

Standard AP and lateral radiographs were used. The Gartland classification of the lateral film was recorded by PHR (Gartland 1959). This classification has good intra- and interobserver reliability (Barton et al. 2001).

### Clinical examination

All patients were given written information about the study and were invited to attend a follow-up clinic. 3 patients had moved abroad or to a different part of the country. 9 patients were lost to follow–up, and 22 patients refused to participate, leaving 78 patients available for clinical examination on average 4.3 (1.5–9) years after admission. The surgeon's experience was not revealed to the investigators examining the patients (PHR, EAS, and IS). The cubitus angle and range of motion were measured clinically using a goniometer. The Flynn criteria (Flynn et al. 1974) were used to assess the functional and cosmetic outcome. Neurovascular status was assessed clinically and the patients completed the Quick-DASH score (www.dash.iwh.on.ca 2009) with the help of their parents. The overall satisfaction of the end-result was assessed using a VAS score where zero was the worst imaginable result and 10 was the best (equivalent to the uninjured arm).

### Statistics

Standard descriptive analyses were performed. The variances in Flynn score and the Quick-DASH score were very small and the distribution was much too skewed to allow further statistical analysis. However, the VAS score could be analyzed statistically, and was the main outcome variable we used to demonstrate the difference between the statistical methods (Table 4).

Initially, an unconditional model (one without explanatory variables) was estimated to calculate the ICC, which can be interpreted as the percentage of variation in VAS score that could be explained by the surgeon level.

A hierarchical linear modeling approach with 2 levels (patient and surgeon) was chosen to model the main outcome variable VAS score. Even though the VAS score was a highly skewed variable, a 2-level linear model (Fitzmaurice et al. 2004) was estimated. Simple analyses were then used to assess the association between the outcome variable and the main predictors. 4 variables were considered to be main predictors for the VAS score. The Gartland classification and the surgeon's level of experience (either consultant or resident) were considered to be predictor variables based on the notions that more displaced fractures have more complications and that more experienced surgeons have better results. The variable night (between midnight and 8 a.m.) or day was selected based on the tendency to perform fewer operations during the night to reduce the risk of adverse outcome (Rothschild et al. 2009). There is no consensus on the number of pins needed for adequate fixation. Some authors recommend the use of 3 pins in unstable fractures (Gordon et al. 2001, Vlahovic and Bumci 2002). We therefore included the number of pins as a predictor variable in the model. Time to surgery, preoperative neurovascular status, age, and sex were considered as confounders.

Technically, to account for interactions a new variable, which is the product of 2 (usually) independent variables, is introduced into the model. One should not try out all possible interactions since this increases the risk of spurious results. Thus, when building the multiple model, we only considered variables that we thought might produce an interaction. We decided that the surgeon's level of experience (consultant or resident) and time to surgery might have different effects on outcome depending on the severity of the fracture. We therefore included these 2 interaction terms in the original multiple model. The R^2^, quantifying the proportion of outcome variation explained, was used for model reduction. Akaike's information criterion was applied for covariance model selection (Akaike 1974). Even though the hypothesis about normally distributed residuals could not be accepted, the qq-plot for residuals demonstrated an almost straight line with some small exceptions in the tails.

## Results

There were no cases of permanent neurovascular injury, deep infection, or compartment syndrome. The retrospective review of the medical records of the 112 fracture patients revealed 21 postoperative complications (19%) (Table 2). Gartland 3 fractures were operated earlier and more often by consultants, and had more complications than Gartland 2 fractures.

**Table 2.** Postoperative complications recorded for the 112 fractures

Type of complication	n (*%*)
Reduced / loss of sensation	10 (9)
Radial	2
Ulnar	6
Median	2
Loss of motoric ulnar nerve	1 (1)
Vascular compromise	0
Compartment syndrome	0
Pin site infection	3 (3)
Deep infection	0
Reoperation	7 (6)
Total complications	21 (19)

11 of the 78 elbows available for clinical examination had a cubitus varus malunion. The mean VAS score was lower in patients who developed cubitus varus (7.7 vs. 9.6) than in patients who had a positive carrying angle (p < 0.001, Mann-Whitney test). 20 patients had a positive Quick-DASH score (Table 3).

**Table 3.** Clinical outcome of 78 fractures at follow-up, mean 4.3 years after surgery

Outcome	n
Cubitus varus	11
Flynn's criteria (cosmetic)	
Excellent	52
Good	13
Fair	2
Poor	11
Flynn's criteria (functional)	
Excellent	67
Good	7
Fair	0
Poor	4
VAS score	
Mean (SD)	9.3 (1.3)
Median (range)	10.0 (2.9–10.0)
DASH score	
Mean (SD)	0.51 (1.53)
Median (range)	0 (0–6.82)

The overall amount of variation in outcome explained by our model was 18%. By estimating the unconditional model, the ICC was calculated to be 0.25; i.e. 25% of the total variance in VAS score could be accounted for by the surgeon level. This suggests that an ordinary least-squares (OLS) regression analysis would probably yield misleading results. The simple analyses did not show any statistically significant relationships between the 4 predictors and VAS score. However, 10–26% of the explainable variation in outcome was explained by the predictors, which was sufficiently high to be able to include all considered predictors in the multiple model (Table 4).

**Table 4.** Differences in coefficients and corresponding p-values between an ordinary least-squares (OLS) regression model without interactions, with interactions, and a two-level model with interactions. Age and one interaction (Gartland classification × time to surgery) were excluded from the model

Variable	OLS model without interactions Coeff. (p-value)	OLS model with interactions Coeff. (p-value)	Two-level model with interactions Coeff. (p-value)
Intercept	10 (< 0.001)	9.9 (< 0.001)	10 (< 0.001)
Surgeon's experience	–0.5 (0.15)	0.04 (0.93)	–0.05 (0.91)
Gartland classification	–0.5 (0.13)	0.7 (0.29)	0.7 (0.27)
Number of pins	0.8 (0.02)	0.8 (0.01)	0.6 (0.07)
Night/day	–0.9 (0.03)	–0.9 (0.02)	–1.0 (0.01)
Time to surgery	0.01 (0.24)	0.02 (0.10)	0.02 (0.14)
Neurovascular status	0.7 (0.19)	0.7 (0.19)	0.7 (0.16)
Gender	–0.3 (0.39)	–0.4 (0.20)	–0.4 (0.20)
Gartland × surgeon's competence	–	–1.5 (0.060)	–1.4 (0.046)

In the OLS model without interactions, there was a positive correlation between number of pins and patient satisfaction (p = 0.02), while in the 2-level model with interaction terms included this correlation was not significant (p = 0.07). Neither the surgeon's experience nor the severity of the fracture influenced the VAS score significantly. The combination (interaction) of the two, with marginally significant effect on the outcome (p = 0.05), influenced the conclusions sufficiently to be included in the final model.

Although the 2-level model corrected the problem of underestimated standard errors in OLS regression, the results did not differ much in the 2 models. Only the number of pins was no longer statistically significant in the 2-level model. If we had used only a simple OLS model, we would have reported number of pins as an explanatory variable.

## Discussion

Our point is not that the last word about treatment of supracondylar fractures has been said, but that simple statistical models do not suffice. We found that the clinical outcome after pinning of displaced supracondylar humerus fractures in children is good or excellent in most cases, which is consistent with previous reports (Foead et al. 2004, Devkota et al. 2008). The few complaints were mainly cosmetic, without loss of function. This is a difficult starting point when searching for factors to improve treatment, and we could only explain 18% of the variance in outcome. Even so, the use of statistical analyses that adhere more closely to reality than a simple OLS model changed conclusions. We have considered the most commonly used explanatory variables for inferior outcome and found that an interaction variable affected conclusions, and that as much as 25% of the explained variance could be attributed to the surgeon level. Hence, we argue that future observational studies in orthopedics must consider inclusion of interactions. Furthermore, since surgery is 2-level in nature (patient and surgeon), the need for a 2-level model should always be explored by calculating the intraclass correlation coefficient (ICC). This is possible even in relatively small materials. We also argue that the level of explainable variance should be stated in published papers, so that readers can consider the clinical relevance of statistically significant results. Furthermore, findings in previous studies where an OLS model without interactions has been used—and where the conclusions have had an impact on clinical practice—should be reviewed using these statistical methods.
